# Estimating the Future Health and Social Care Costs of Alzheimer's Disease Dementia in the UK: Impact of Disease Modifying Therapy Efficacy, Uptake, and Care Model – A Scenario Modelling Study

**DOI:** 10.1002/gps.70185

**Published:** 2026-02-15

**Authors:** Marc Evans, Craig Ritchie, Dominic Trepel, Julie Hviid Hahn‐Pedersen, Jamie Kettle, Mei Sum Chan, Benjamin D. Bray, Alice Clark, Milana Ivkovic, Christian Ahmad Wichmann, Sophie Edwards

**Affiliations:** ^1^ University Hospital Llandough Wales UK; ^2^ Scottish Brain Sciences Gyleview House Edinburgh UK; ^3^ University of St Andrews College Gate UK; ^4^ Trinity College Dublin School of Medicine Trinity Biomedical Sciences Institute Trinity College Dublin Ireland; ^5^ Novo Nordisk A/S Søborg Denmark; ^6^ Health Analytics Lane Clark & Peacock LLP London UK; ^7^ Central and North West London NHS Foundation Trust London UK

**Keywords:** access, Alzheimer's disease dementia, care model, disease‐modifying therapy, healthcare cost, social care cost

## Abstract

**Background:**

To model scenarios exploring potential impacts of disease‐modifying therapies (DMTs) for Alzheimer's disease (AD) dementia on future health and social care costs in the United Kingdom.

**Methods:**

A cohort Markov model was developed using population projections and published AD epidemiological data. Stage‐specific transition rates (mild cognitive impairment due to AD and mild, moderate, severe AD dementia) and health and social care cost data were applied to estimate cost outcomes over 2020–2040. Potential proportion of eligible population receiving treatment (uptake) and follow‐up care models (primary vs. specialist care) were elicited from expert opinion. Scenarios combined ranges of DMT efficacy estimates, uptake, and care model. DMT price was excluded due to no UK precedent.

**Results:**

Without DMT access, 1,038,405 people (1.5%) were projected to have AD dementia by 2040. Under the various DMT treatment scenarios, the prevalence of AD dementia by 2040 was projected to be 34,000–98,000 cases lower. Associated cumulative cost offsets were higher, £4.4–12.9billion over 2020–2040, in scenarios where most individuals received primary care follow‐up, compared with majority specialist care follow‐up (‐£2.3billion to +£3.2billion). Assuming DMT efficacy of 25%, 58% uptake and majority primary care follow‐up cumulative cost offsets increased from £4.4billion to £10.1billion by 2040 but the UK Health Service would need to diagnose and provide DMT for over a million individuals by 2030 and two million by 2040 to achieve this.

**Conclusions:**

Potential cost offset from DMT are large but highly dependent on the model of healthcare delivery and the ability of healthcare systems to scale up diagnosis and treatment services.

## Key Points

- Previous modelling of disease‐modifying therapies (DMTs) in Alzheimer's disease (AD) dementia estimated economic benefits using various estimated DMT efficacies but did not consider how delivery care model and/or uptake might influence long‐term cost savings.
- Hypothetical DMTs were estimated to yield potentially large reductions in the cost of health and formal social care, leaving aside the cost of the treatments themselves, by reducing the number of people progressing to more severe stages of dementia. Cost offsets begin to accelerate approximately 5 years after DMTs becomes available.
- The size of these cost offsets depended particularly on the speed and scale of the adoption of DMTs into routine clinical practice and the model for treatment and follow‐up (e.g., having a majority primary‐care model for follow‐up).
- Early diagnosis and healthcare system preparedness to implement diagnosis, treatment and follow‐up pathways at the necessary scale, will be important factors in the potential for DMTs to reduce the Alzheimer's disease dementia burden on the cost of health and social care.

## 1 Introduction

Approximately 900,000 individuals aged 65 years or over are currently living with dementia in the United Kingdom [1]. Driven by an ageing population, this is projected to increase to around 1.6 million in 2040 alongside a sharp rise in the cost of dementia care [1]. Dementia has many impacts on affected individuals, their families, and society. In 2019, the total cost of dementia to the UK was £34.7 billion per year, which equated to approximately £32,250 per person with dementia per year [1]. Dementia care incurs healthcare, social care (home and residential care), and unpaid care costs. The largest proportion of cost is attributed to social care, which is estimated to total £15.7 billion and of which more than 60% is paid by people with dementia and their families. In addition, families and friends provide unpaid care to people with dementia to a value of £13.9 billion per year [1]. The time spent on delivering care and the associated impact on care partners' health and wellbeing are significant and often overlooked. It is estimated that informal care partners spend 1.1 billion hours each year caring for individuals with dementia [2]. Only 18% of care partners are in paid employment, reflecting wider indirect costs to society [3].

Alzheimer's disease (AD) dementia is the most common type of dementia, accounting for 60%–70% of all dementia diagnoses [4]. The severity of AD is generally classified on a spectrum from mild cognitive impairment (MCI) due to AD through to severe AD dementia. MCI is a heterogenous condition which reflects a decline in cognitive function distinct from ageing with minimal impairment in activities of daily living [5, 6, 7]. MCI due to AD is defined as the presence of MCI and of specific AD biomarkers including *β*‐amyloid and phosphorylated tau [8]. Individuals with MCI due to AD will progress to AD dementia [9, 10].

Existing treatments for AD dementia (i.e., cholinesterase inhibitors and memantine) aim to relieve symptoms. In recent years a new treatment paradigm for AD has emerged in the form of disease‐modifying therapies (DMTs) which aim to slow down disease progression. Clinical trials of DMTs in individuals with MCI due to AD and those with mild AD dementia are reported to slow disease progression [11, 12]. In the US, one monoclonal antibody based therapy that targets *β*‐amyloid proteins and reduce amyloid plaques in the brain has been approved [13]. Although there are currently no DMTs for AD with marketing authorisation in Europe or the UK, DMTs may also become available as a treatment option in Europe in the next few years.

DMTs will potentially lead to large changes in how individuals with MCI due to AD and mild AD dementia are diagnosed and treated, and could help to reduce the very large burden including costs to patients and health systems from this disease [14, 15, 16]. We therefore aimed to model the potential impact of DMTs on future health and formal social care costs of AD dementia in the United Kingdom, exploring a range of scenarios including treatment efficacy, uptake (proportion of treatment eligible population receiving treatment), and care model. The results of this study could help health systems prepare and plan for the implementation of DMTs.

## 2 Methods

### 2.1 Study Design

This scenario modelling study was conducted using a Markov model, which operates under several key assumptions: it is memoryless (the future state depends only on the current state), calendar time‐homogeneous (transition probabilities are identical for all individuals in a given state and remain constant over time), comprehensive (incorporating all relevant states of disease progression), and mutually exclusive (each state is distinct with no overlap) [17]. Model parameters were extracted from published literature following a targeted literature review [1, 6, 18, 19, 20, 21, 22, 23, 24, 25, 26, 27, 28], and the opinion of four clinical and academic experts was sought to estimate parameters relating to future care models and uptake (since DMTs were not part of clinical practice outside of clinical trials at the time of writing).

### 2.2 Model Structure

A cohort Markov model with annual cycles was developed to predict the distribution of the UK population across four stages of AD (MCI due to AD, and mild, moderate, severe AD dementia) over a 20‐year time horizon (between 2020 and 2040). Projected population distributions were used to derive estimates of expected healthcare, formal social care, and treatment costs. A baseline scenario of no access to DMT was projected [21] (where “no access to DMT” was defined as usual care including treatment with symptomatic dementia treatments when indicated) followed by scenarios with access to DMT where outcomes were modelled on three key parameters: DMT efficacy, uptake, and care model.

### 2.3 Model Inputs

#### 2.3.1 Parameters for Baseline Scenario

**FIGURE 1:**
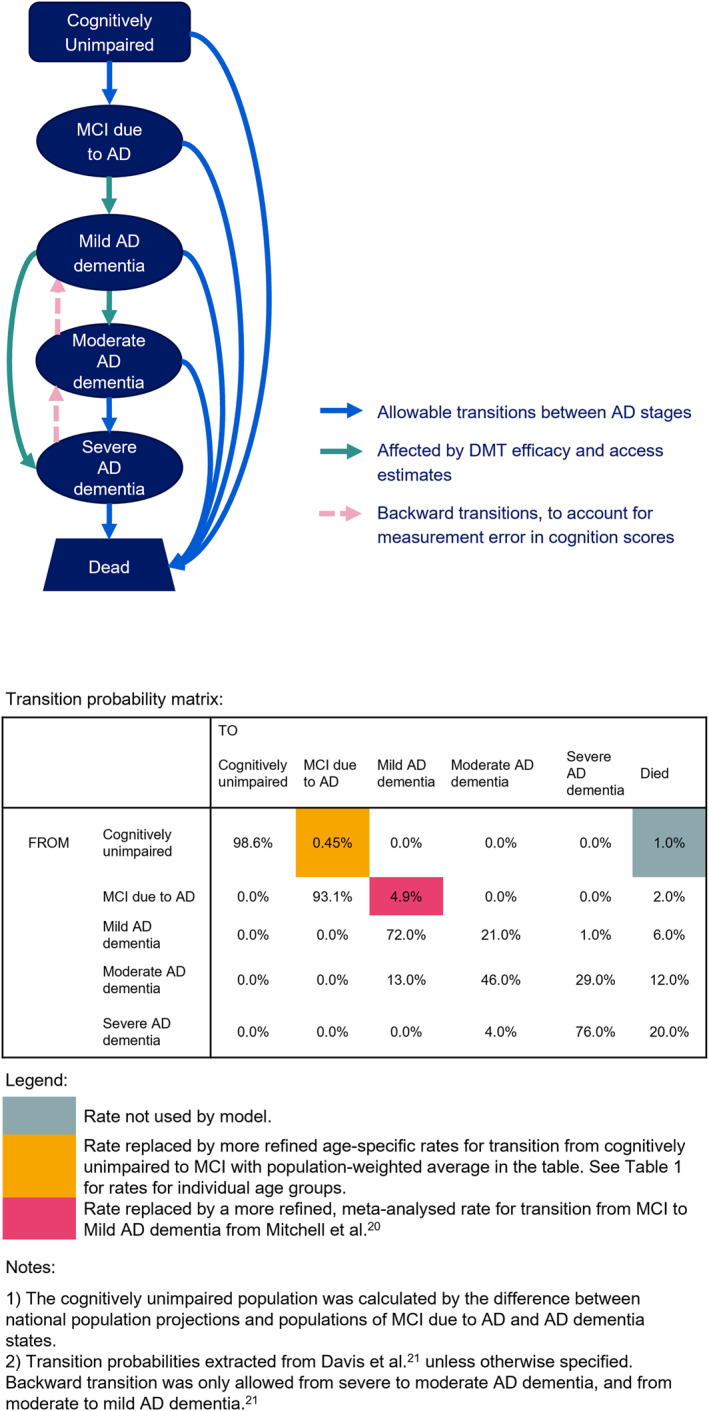
State structure for the cohort Markov model with allowable transitions (yearly rates) between states.

State structure of the cohort Markov model and transition rates are illustrated in Figure 1. National population and changes in age distribution were based on projections from the Office for National Statistics [29]. Prevalence of AD dementia (Table 1), proportion of individuals with AD dementia by disease severity (Table 2) and prevalence of MCI due to AD (Table 1) by age group at baseline were informed by published literature with extrapolation (see Supplementary Methods, Supporting Information S1). Yearly transition rates including mortality rates (Figure 1) were assumed to be the same for all age groups, except for incidence of MCI due to AD which increases with age (Table 1, Supporting Information S1). Backward transitions from severe to moderate and moderate to mild AD dementia were allowed to account for misclassification of AD dementia staging (Figure 1). The cognitively unimpaired population was calculated by the difference between national population projections and populations of MCI due to AD and AD dementia states.

**TABLE 1:** Key baseline parameters: (1) AD dementia baseline prevalence, (2) MCI due to AD baseline prevalence and incidence rates, (3) total per person per year healthcare resources utilisation (HCRU) costs for all England and (4) total per person per year HCRU costs for all AD, by age group.

	AD dementia		MCI due to AD		All England [23, 24, 25]		All AD [26]	
	Baseline prevalence		Baseline prevalence	Incidence	Total per person per year HCRU costs		Total per person per year HCRU costs	
Age	Male	Female	All (male and female)		Male	Female	Male	Female
30–34	0.0001	0.0002	0.0039	0.0001	£693.86	£1789.63	£2626.96	£2626.96
35–39	0.0003	0.0003	0.0054	0.0002	£734.49	£1515.61	£2626.96	£2626.96
40–44	0.0005	0.0005	0.0075	0.0003	£821.90	£1268.05	£2626.96	£2626.96
45–49	0.0009	0.0010	0.0103	0.0005	£1045.25	£1364.51	£2626.96	£2626.96
50–54	0.0016	0.0018	0.0143	0.0009	£1222.83	£1502.76	£2740.08	£2740.08
55–59	0.0028	0.0033	0.0197	0.0015	£1448.22	£1615.18	£2996.39	£2996.39
60–64	0.0052	0.0059	0.0297	0.0024	£1663.87	£1698.39	£3327.09	£3327.09
65–69	0.0078	0.0117	0.0416	0.0043	£2412.88	£2221.96	£3740.76	£3740.76
70–74	0.0195	0.0163	0.0553	0.0078	£2795.88	£2479.90	£4019.29	£4019.29
75–79	0.0338	0.0403	0.0886	0.0135	£3621.93	£3027.38	£4130.35	£4130.35
80–84	0.0689	0.0618	0.1630	0.0265	£4181.89	£3505.93	£4643.33	£4643.33
85–89	0.0832	0.1177	0.2612	0.0417	£4781.36	£3891.96	£5001.80	£5001.80
90–94	0.1112	0.2275	0.2757	0.0745	£4797.56	£3461.62	£5547.85	£5547.85
95–99	0.1112	0.2275	0.2757	0.1218	£4548.80	£3219.13	£5460.43	£5460.43
100 & over	0.1112	0.2275	0.2757	0.1990	£3218.98	£2088.91	£4465.27	£4465.27
Aggregated	0.0334 (age standardised 65+)	0.0484 (age standardised 65+)	0.1009 (age standardised 65+)	0.0045 (population weighted)	—	—	—	—

Abbreviations: AD: Alzheimer's disease; HCRU: healthcare resources utilisation; MCI: mild cognitive impairment.

**TABLE 2:** Key baseline parameters: (1) baseline AD dementia severity proportion split by age group (excluding MCI due to AD) and (2) ratio of costs by AD dementia severity to the all‐severity AD dementia cost.

	Mild AD dementia	Moderate AD dementia	Severe AD dementia	Total AD dementia
Baseline AD dementia severity proportion split by age group (excluding MCI due to AD) [1]				
35–64	58.82%	35.29%	5.88%	100.00%
65–74	17.92%	40.83%	41.25%	100.00%
75–84	18.97%	40.18%	40.84%	100.00%
85+	14.70%	31.53%	53.78%	100.00%
Ratio of costs by AD dementia severity to the all‐severity AD dementia cost [28]				
Health care	0.5548	1.4829	1.7597	1
Social care	0.4261	1.7049	1.7622	1

Abbreviations: AD: Alzheimer's disease; MCI: mild cognitive impairment.

#### 2.3.2 Key Treatment Efficacy, Uptake, and Care Model Parameters Influencing AD Populations

In scenarios with DMT, DMTs were assumed to be approved in the UK in 2025 to treat people who have either MCI due to AD or mild AD dementia. Input from clinical and scientific experts was sought to define plausible values of key parameters for DMT adoption into clinical practice and treatment strategies, including future care models. A numerical mean was applied to plausible ranges and most likely values of uptake, as well as the proportion of patients treated under each care model based on expert opinion. Additionally, the time (in years) required to achieve high uptake was calculated as a weighted mean. What‐if scenarios were simulated for the population of MCI due to AD and AD dementia as well as annual total care costs using defined values for each parameter, provided in Table 1. Consolidated expert responses were provided in Supporting Information S1.

In scenario analyses, using a DMT was assumed to slow disease progression from the MCI and mild AD dementia stages to the more severe AD stages (Figure 1) by 20% (low), 25% (medium) and 30% (high). Modelled DMT efficacies were based on published efficacies of 22%–36% from DMTs undergoing clinical trials in AD which used surrogate endpoints [11, 12]. Efficacy estimates were reviewed by clinical and scientific experts and agreed by majority vote (Supporting Information S1). Two uptake scenarios were modelled based on the assumption that uptake of DMT for the treatment eligible population increases steadily between 2025 and 2034: a low‐uptake scenario (uptake proportion increases from 0% to 25%) and a high‐uptake scenario (uptake proportion increases from 0% to 58%). The care model of DMT was defined to be either majority primary care follow‐up or majority specialist care follow‐up (Table 3). Both models involved additional healthcare visits related to the administration of DMT and follow‐up that would not already be captured in the AD state health costs. While both models consisted of an initial specialist appointment, they contributed to different healthcare costs the subsequent visits in primary care versus specialist care.

**TABLE 3:** Key treatment parameters.

DMT efficacy in delaying AD progression
High: 30%
Medium: 25%
Low: 20%
DMT uptake
High: Increasing from 0% to 58% between 2025 and 2034
Low: Increasing from 0% to 25% between 2025 and 2034
Care model
Majority primary care follow‐up: 4 primary care follow‐up visits per year
Majority specialist care follow‐up: 2 primary care follow‐up visits and 2 specialist care follow‐up visits per year

Abbreviations: AD: Alzheimer's disease; DMT: disease modifying therapy.

Core scenarios with DMT access include three high‐uptake scenarios for each of the efficacy levels (high, medium, and low), and one scenario for low uptake and medium efficacy.

#### 2.3.3 Healthcare, Social Care, and Treatment Costs

Costs were calculated based on healthcare, social care, and DMT‐related follow‐up appointments to capture expenses from a societal perspective. Healthcare costs were defined as healthcare resources utilisation (HCRU) costs per person per year in England and for the full AD dementia population (Table 1 [23, 24, 25, 26]) stratified by age group and sex. Similarly, formal social care costs were identified nationwide and for the AD dementia populations assuming 48% of the AD dementia population utilised formal social care. Nationwide formal social care costs were £200.26 for people aged 18–64 and £499.89 for those aged 65 and above [27]. Social care costs for all AD dementia population were £12,526 for males and £12,480 for females [26]. Healthcare costs and social care costs for the AD dementia population were multiplied by ratios of costs by AD dementia severity (Table 2). Additional treatment costs associated with DMT were based on costs of specialist care for AD dementia taken from published literature [30], which reported a cost of £465 per NHS memory clinic visit and £33 per primary care visit. The cost of DMTs in the UK were not known at the time of analysis therefore a secondary analysis was conducted exploring DMT annual costs of £1,200, £11,100 and £21,000 by 2040. The ranges were chosen to reflect costs of repurposing existing treatments at the lower end and likely costs of newer DMTs at the upper end. A recent lecanemab assessment by NICE indicated that yearly treatment costs are in the region of £19,000 per person per year of treatment (i.e., £545 for 500 mg solution; with the total cost being dependent on patient body weight) [31].

Future costs were not discounted. Full cost equations were reported in the Supplementary Methods. Only potential changes in healthcare practice due to DMT for AD were considered in this model. Any other changes which may occur between 2020 and 2040 for other disease areas were not modelled. As a result, we assumed the average annual health and social care spend per person without AD and at each stage of AD dementia stayed at current levels.

### 2.4 Model Analysis

Modelling was conducted using Microsoft Excel. Yearly populations of all model states were generated from 2020 – 2040 for the baseline scenario using national population projections, baseline prevalence estimates, and transition probabilities (Supplementary Methods). The populations were then used to compute respective estimates of expected healthcare, social care, and treatment costs. The impact of DMT on AD dementia prevalence was estimated across the range of efficacy and uptake levels in DMT scenarios. The projected populations in DMT scenarios and the care model were then used to inform the annual and cumulative cost estimations. Further scenario analyses were performed using medium DMT efficacy with majority primary care follow‐up, see Supplementary Methods and Table S2. Costs were reported as British pounds in 2019 monetary values to mitigate fluctuations related to the pandemic. Results were reported in line with the CHEERS 2022 checklist in Supporting Information S1.

## 3 Results

**FIGURE 2:**
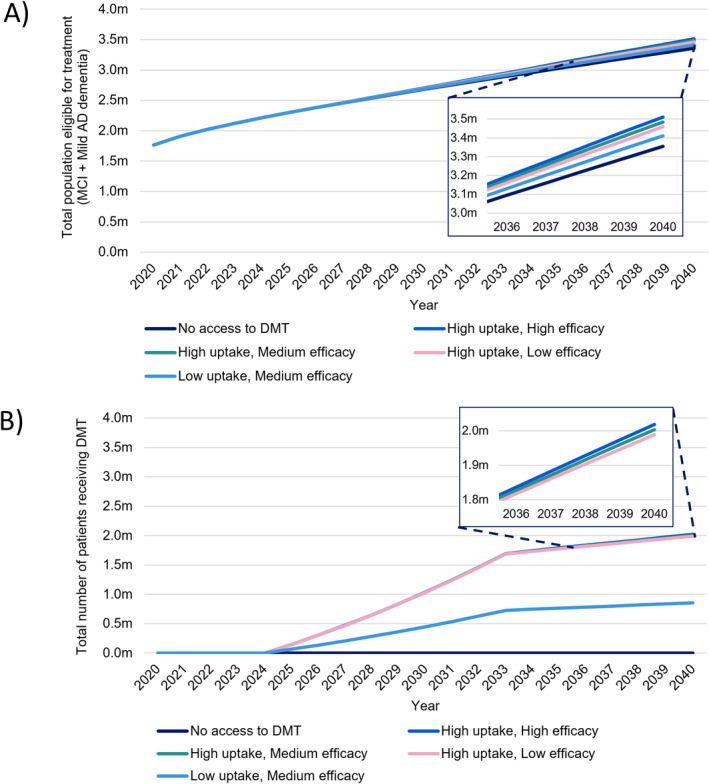
Total population (A) eligible for and (B) receiving disease modifying therapy (DMT) from 2020–2040; irrespective of care model.

In the baseline scenario with no DMTs, 1,038,405 people (1.5% prevalence) were projected to have AD dementia in the UK by 2040, compared to 570,212 people (0.9% prevalence) in 2020, an 82% increase (Table 4). The population prevalence of AD dementia was projected to increase by 0.6%–1.5% by 2040. 2,804,190 (4%) people were projected to have MCI due to AD in the UK by 2040, compared to 1,648,518 (2%) in 2020. The total population eligible for treatment (MCI due to AD population, and mild AD dementia population) was projected to increase from 1,766,428 in 2020 to 3,355,730 in 2040, with subtle differences in temporal trends across scenarios (Figure 2A & Table S3 in Supporting Information S1).

**TABLE 4:** Estimated number of people with a diagnosis of AD dementia, change in prevalence of AD dementia, and change in annual cost compared to no access to DMT from 2020–2040 in majority primary care scenarios.

Year	2020	2025	2030	2035	2040
Estimated number of people with a diagnosis of AD dementia in the UK					
No DMT	570,212	634,067	770,603	909,007	1,038,405
High uptake, high efficacy	570,212	634,067	746,417	836,875	940,819
High uptake, medium efficacy	570,212	634,067	750,468	849,168	957,862
High uptake, low efficacy	570,212	634,067	754,511	861,351	974,585
Low uptake, medium efficacy	570,212	634,067	761,873	883,319	1,004,319
Change in prevalence of AD dementia compared to no access to DMT					
High uptake, high efficacy	0	0	−24,186	−72,132	−97,586
High uptake, medium efficacy	0	0	−20,135	−59,839	−80,543
High uptake, low efficacy	0	0	−16,092	−47,656	−63,820
Low uptake, medium efficacy	0	0	−8730	−25,688	−34,086
Change in annual cost compared to no access to DMT					
High uptake, high efficacy	£0.0bn	£0.1bn	‐£0.3bn	‐£1.3bn	‐£1.9bn
High uptake, medium efficacy	£0.0bn	£0.1bn	‐£0.2bn	‐£1.0bn	‐£1.6bn
High uptake, low efficacy	£0.0bn	£0.1bn	‐£0.1bn	‐£0.8bn	‐£1.2bn
Low uptake, medium efficacy	£0.0bn	£0.0bn	‐£0.1bn	‐£0.4bn	‐£0.7bn

Abbreviations: AD: Alzheimer's disease; DMT: disease modifying therapy.

As a result of slowing disease progression, DMTs are estimated to modestly reduce the prevalence of AD dementia, with the total AD dementia population reducing by between 34,086 and 97,586 by 2040 depending on DMT efficacy and uptake (Table 4). The total population eligible for treatment was projected to range from 3,411,617 to 3,510,868 in 2040. The number of people treated with DMTs varied under the different uptake scenarios, but in the high uptake scenarios, the models estimated that approx. 146,709 people would be treated in 2025, rising to 2,018,749 by 2040 (Figure 2B and Table S3).

**FIGURE 3:**
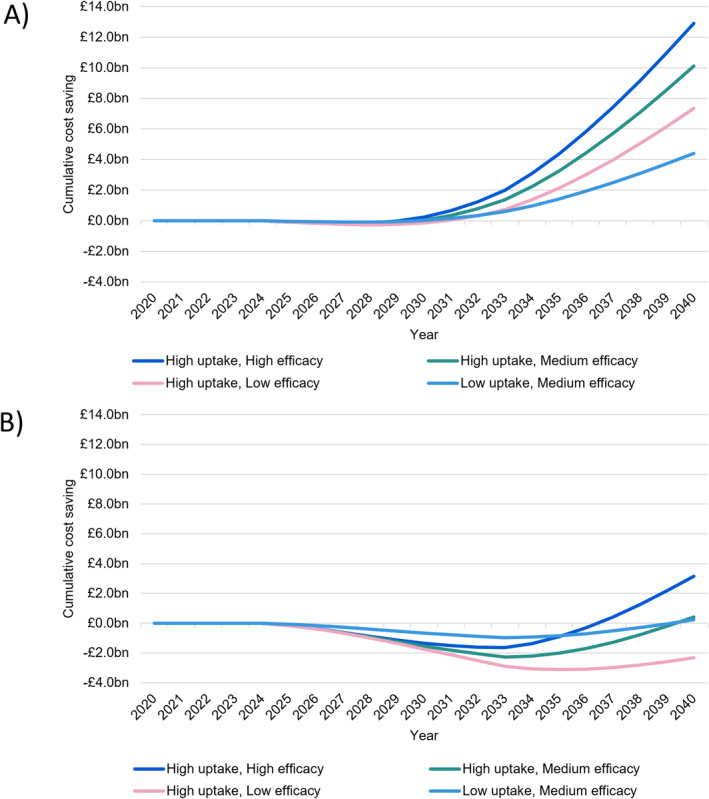
Cumulative cost offset by 2040 versus no disease modifying therapy (DMT) with (A) majority primary care or (B) majority specialist care follow‐up.

**FIGURE 4:**
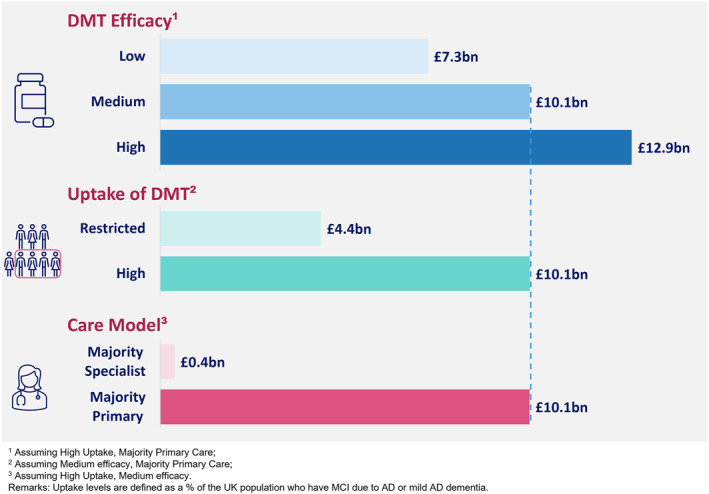
Cumulative health and social care cost offsets by 2040 versus no disease modifying therapy (DMT).

Total cost offsets from healthcare, social care, and treatment were as a result of slowing progression to more severe AD dementia stages, which were associated with higher costs, given that costs of the DMT were not modelled in this study. In the scenarios with majority primary care follow‐up, moving from low uptake to high uptake raised cumulative cost offsets from £4.4 billion to £12.9 billion by 2040 (Figures 3A and 4). This was equivalent to £352 to £614 annual cost offsets per treated patient by 2040. Projected offsets were much lower with a majority specialist care model, where projected cumulative cost offsets ranged from ‐£2.3 billion to £3.2 billion by 2040 in core scenarios (£0.4 billion by 2040 was projected in a high uptake‐medium efficacy scenario, Figures 3B and 4). Costs saved per patient year treated were ‐£112 to £150, ∼£450 lower than the equivalent scenarios with majority primary care. Regardless of care model being majority primary or majority specialist, increased costs were observed in beginning years of DMT availability due to the time lag before treatment benefits manifest in terms of delayed AD progression. This was due to the cost of providing initial specialist care appointments when commencing DMT and would be higher if the costs of the DMT itself were included.

Scenario analysis modelling extreme parameters based on medium efficacy (25%) showed a broader range of cumulative cost offsets. In the extreme low scenario where uptake was 10%, a cumulative cost offset of £1 billion was projected by 2040 compared to no access to DMT (Figure S4). The extreme high combination demonstrated the potential impact of having almost immediate access to DMT for the whole eligible population and full uptake in one year, with a cumulative cost offset of £25.8 billion by 2040 compared to no access to DMT (Figure S4).

The secondary analysis indicated that, in high uptake scenarios, annual costs for DMTs in 2040 could range from £2.4 billion for lower‐cost options to £41.8–£42.4 billion for higher‐cost therapies, irrespective of the treatment's efficacy. In scenarios with low uptake and medium efficacy, annual costs for DMTs were substantially lower, ranging from £1.0 billion to £17.9 billion (Table S4).

## 4 Discussion

This scenario modelling study provides estimates of the longer‐term impact of DMT on the healthcare and cost burden of AD dementia in the UK considering a range of DMT efficacies, uptake and delivery care model. Firstly, due to population ageing, we estimate that the number of people with AD dementia in the UK will almost double (to approximately 1 million people) by 2040. DMTs were estimated to modestly reduce the number of individuals with AD dementia but yield potentially large offsets in the cost of health and social care (£4.4 to £12.9 billion over 2020–2040) by slowing progression to more severe stages of AD dementia where health and social care needs are larger. Adjusted for inflation this amounts to £4.9 to £14.3 billion in 2023 [32]. Secondly, the size of these cost offsets was dependent to some extent on DMT efficacy but more substantially by the proportion of eligible individuals treated with DMTs and the model of healthcare delivery for treatment initiation and follow‐up. Finally, our estimates suggest that the National Health Service in the UK will need to diagnose and provide DMT for over a million individuals by 2030 and two million by 2040 to achieve high uptake (58%) and yield the largest health and social care offsets estimated in these scenarios. This scaling up of specialist AD diagnosis and treatment services is potentially a major challenge requiring health system preparedness for the implementation of DMT at scale.

The care model made a major difference to future costs, with significantly smaller projected offsets in majority specialist care scenarios. The decrease in cost offsets compared to majority primary care was due to the higher cost of specialist care appointments compared to primary care (£465 vs. £33 per visit). Given the monitoring required for current DMTs involves radiographic monitoring and specialist assessment, the costs associated with specialist care in this study are likely to be conservative. We note that the DMT care model is likely to be driven by therapy specific requirements and associated clinical considerations.

Differences in the uptake scenarios made a larger proportional difference to cost offsets than differences in estimated DMT efficacy. Achieving optimum uptake of DMTs in treatment‐eligible populations is likely to be key for health systems to realise these large potential offsets in healthcare cost in the long term. The population with MCI due to AD is large (although there is more uncertainty about the prevalence of this compared to AD dementia), younger than the AD dementia population and does not benefit from current treatment options available. Given the large number of individuals eligible for DMT treatment, experts have highlighted that there will be challenges for the healthcare system to provide timely and equitable access to DMTs [33].

### 4.1 Comparison With Existing Literature

Our AD dementia population projections in the baseline scenario are in line with published projections of ∼1.4 m in UK in 2051 [28], as well as a published estimation of the AD dementia population doubling every 20 years with current treatment options [34]. A recent analysis projected that the all‐cause dementia population in England and Wales would reach 1.7 million (1.62 – 1.75 million) by 2040, assuming a 2.8% relative annual increase in incidence rate [35]. If 65% of dementia is due to AD [36, 37], this would be equivalent to an AD dementia population of 1.1 million (1.05 – 1.14 million) in England and Wales. This estimate is in line with our projected AD dementia population (1 million) for England. In the long run, AD dementia population could be affected by changes in incidence related to underlying risk factors such as cardiovascular disease and obesity.

We modelled the potential impact of DMTs on the burden of AD dementia using DMT efficacy, level of uptake of DMTs and care model. The projected DMT efficacy levels we investigated (20%–30%) are slightly more conservative than the reported efficacy levels of DMTs 22%–36% [11, 12]. The scenarios with more conservative DMT efficacy and higher update could simulate potentially lower real‐world effectiveness associated with provision of DMT beyond trial populations as discussed in the literature [38].

Our estimates of health and social care cost offsets are comparable to other published estimates in the UK, although there is between‐study variation in the assumptions and data sources used. Average annual cost offsets of £0.6 billion (£614 per patient year treated) for the high uptake and high treatment efficacy scenario are lower compared to other published estimates [39] of £1.0–1.6 billion (£743–791 per patient year treated, if we assume an average life expectancy for a 70‐year old with MCI due to AD of 13.8 years [40]) for broadly similar full access treatment scenarios. When extrapolated to lifetime cost offsets per treated patient, the projected offsets in this study would be £8,480, compared to published estimates of £10,260–10,910 [39]. A recent US study estimated larger cost offsets of $0.783 to $3.132 trillion from 2022 to 2050 based on a delay of onset of AD dementia by 5 years and various uptake scenarios, reflecting differences in prevalence and health systems between the UK and US [41]. Informal care costs for people with MCI due to AD were not estimated due to lack of data. If informal care costs for people with AD dementia were included, baseline projected costs could increase to £3010 billion (8.5% increase) by 2040 [39] (including £296 billion in health and social care costs and £237 billion in informal care costs for people with AD dementia). Furthermore, slowing dementia progression will likely manifest wider societal and cost benefits to individuals with AD dementia, their families, and carers.

### 4.2 Limitations

Differences in health systems could impact study findings, as we focussed on potential implementation in a UK healthcare setting. Health system differences that may impact costs include drug approval, market access, reimbursement agreements and clinical settings, all of which vary significantly between countries [42, 43, 44, 45]. Modelling the impact of inequity in access to DMTs and of newer generations of DMT with potentially different routes of administration [46], although important for assessing the full potential benefit of DMTs, was outside the scope of this study.

Our projection model aimed to understand the average impact of DMT at a population level, given a lack of individual data. However, this approach was chosen over microsimulation where data on the probability of transitions was unavailable to reflect individual‐level transitions. Differential efficacy of DMT by sex was not performed, instead the model incorporated weighted average efficacies at a population level. The model depended on expert opinion for some parameters regarding future care models and uptake ranges, and the availability of published epidemiological and cost assumptions. Currently there are no AD DMT precedents used in clinical practice to inform these. However, the model offered a framework where emerging evidence for key treatment parameters, such as DMT efficacy, DMT cost and informal care costs, to be incorporated.

Published assumptions account for measurement error in AD transitions (e.g., including backward transitions to less severe AD dementia stages), and might not reflect reversal or improvement of the functional impairment that defines the dementia stages in the real world. AD transition estimates that consider clinically misclassified AD dementia stages [47] were not in a suitable format to input into the model.

With the model design, all individuals in the MCI due to AD and mild AD dementia states who were part of the uptake received DMT for as long as they were eligible, including those who transitioned backward to mild AD dementia state. This was hypothetical and might not reflect the clinical practice or treatment duration recommendations which were not available at the time of writing.

Fixed transition rates were used except for the transition to MCI due to AD, as transition by age within a specific AD state was not available in literature. All‐cause mortality rates for each of the non‐baseline states from Davis et al. [22] were the best available estimations available for this study though they have limitations. The transition rates were based on the National Alzheimer's Coordinating Centre Uniform Data Set which was from the US. Transition rates in other populations could be higher or lower due to underlying differences in demographics, such as age distribution.

A recent modelling analysis to quantify slowing of disease progression in Alzheimer's disease due to DMTs reported that the delays to disease progression increased life expectancy without extending time in severe stages of disease [48]. Our model transitions will have captured the additional time spent in MCI due to AD and early stages of dementia but given the 20 years horizon may not have fully captured the additional burden of these individuals eventually moving to more advanced stages prior to death.

The intention of this model was to estimate the factors driving cost offsets for DMTs generally, rather than to consider specific DMT. The analysis of drug costs is speculative and should be interpreted with caution, since the study was not designed as a cost‐effectiveness analysis and does not account for the broader health and societal benefits of patients remaining in early AD stages for longer periods. The model can be updated in the future, once new DMTs are licensed, to provide more accurate estimates of potential cost offsets for specific therapies.

Costs related to increasing the capacity for diagnosis (e.g., capital expenditure on diagnostic equipment), the administration of DMTs and DMTs' potential side effects were not included in the modelling due to lack of data and because the intention was to develop a model which would be agnostic to the class/mechanism of action of DMT (and hence side effect profile). We recommend that these are included in future modelling of specific DMTs as they are likely to materially impact the cost offsets of specific DMTs, resulting in lower cost offsets than those reported in this study. On the other hand, informal care costs and out‐of‐pocket costs for social care were also excluded due to a lack of data, which likewise should be considered for inclusion in future models when data becomes available, as they are likely to increase the magnitude of the cost offsets as individuals spend more time in earlier dementia stages [49].

Pre‐pandemic costs were used for modelling as these were the latest costs available at the time of the study. The potential limitations of using pre‐pandemic costs for projections in the post‐pandemic setting were acknowledged during the review of model parameter estimates by experts.

### 4.3 Conclusion

The potential health and social care cost offsets from DMTs are large and begin to accelerate approximately 5 years after DMTs become available as a treatment option. Improving uptake to DMTs in the eligible population and having a majority primary‐care model were projected to make larger differences to future costs, compared to differences in projected treatment efficacy. These results indicate that high DMT uptake levels could substantially reduce the burden on individuals and healthcare systems and emphasise the importance of early diagnosis and healthcare system preparedness.

## Author Contributions

SE, DT, CR and ME conceptualisation, interpretation of data, writing – review and editing. JHHP conceptualisation, methodology, writing – review and editing. JK formal analysis, methodology, writing – review and editing. MSC conceptualisation, formal analysis, methodology, writing – review and editing. BDB conceptualisation, methodology, data verification, writing – review and editing. AC, MI, CAW and WM methodology, validation, writing – review and editing. All authors had full access to all the data in the study and take responsibility for the integrity of the data. JK, MSC, BDB had full access to the data and verified the accuracy of the data analysis. All authors contributed to the critical revision of the manuscript and approved the final version of the manuscript for publication. The corresponding author attests that all listed authors meet authorship criteria and that no others meeting the criteria have been omitted. Medical writers (Alejandra Castanon and Florence Ma) contracted by the sponsor provided assistance in preparing the manuscript. Academic authors were invited *pro bono* to provide critiques on the analyses, data interpretation, and manuscript and received no funding for their input.

## Funding

Novo Nordisk funded LCP to conduct the study and participated in study design; analysis and interpretation of data; writing of the report; and decision to submit the article for publication. Medical writers were funded by Novo Nordisk. Academic authors were invited *pro bono* to provide critiques on the analyses and received no funding for their input.

## Ethics Statement

No ethical approval was required. This study draws from open data sources of aggregate data.

## Transparency Declaration

The lead author (the manuscript's guarantor) affirms that the manuscript is an honest, accurate, and transparent account of the study being reported, that no important aspects of the study have been omitted, and that any discrepancies from the study as planned have been explained.

## Conflicts of Interest

SE reports no conflicts of interest. DT is associate editor for Alzheimer’s and Dementia. CR is CEO and founder of Scottish Brain Sciences and reports personal fees from Actinogen, Biogen, Cogstate, Eisai, Eli Lilly, Janssen Cilag, Merck, Novo Nordisk, Roche Diagnostics and Signant Health outside of the submitted work. JK, MSC, BDB were employed by Lane Clark & Peacock LLP (LCP) and BDB is a partner at LCP. CJHHP, AC, MI, WM and CAW were employed by Novo Nordisk who has two ongoing phase 3 trials in early AD and are shareholder of Novo Nordisk A/S. MI also reports shares in Elli Lilly. ME reports personal fees from NN, BI, AstraZeneca, Moderna outside of the submitted work.

## Supporting information


Supporting Information S1

## Data Availability

The data that support the findings of this study are available from the corresponding author upon reasonable request.
