# Supplementary material for: Estimating the Future Health and Social Care Costs of Alzheimer's Disease Dementia in the UK: Impact of Disease Modifying Therapy Efficacy, Uptake, and Care Model – A Scenario Modelling Study
Source: Int J Geriatr Psychiatry. 2026 Feb 15;41(2):e70185. doi: 10.1002/gps.70185 (PMC12907742; doi:10.1002/gps.70185)
Supplement: Supplementary file 1 — Supporting Information S1 [file GPS-41-e70185-s001.docx]

Online Resource

Contents

[**Online Resource methods** 2](#_Toc213423597)

[Parameter calculations 2](#_Toc213423598)

[Cost Equations 2](#_Toc213423599)

[Model inputs and analysis – further information 3](#_Toc213423600)

[**Online Resource figures** 4](#_Toc213423601)

[Figure S1. Dementia prevalence fitting for A) males and B) females^*^ 4](#_Toc213423602)

[Figure S2. MCI due to AD prevalence fitting^†^ 5](#_Toc213423603)

[Figure S3. MCI due to AD incidence fitting^‡^ 5](#_Toc213423604)

[Figure S4. Cumulative cost savings by 2040 versus no DMT in sensitivity analyses 6](#_Toc213423605)

[**Online Resource tables** 7](#_Toc213423606)

[Table S1. Consolidated expert responses 7](#_Toc213423607)

[Table S2. Parameters for further scenario analysis 8](#_Toc213423608)

[Table S3. Total AD population eligible for DMT and receiving DMT from 2020-2040; irrespective of care model 8](#_Toc213423609)

[Table S4. Annual costs of DMT in 2040 in majority primary care scenarios. 8](#_Toc213423610)

[CHEERS 2022 Checklist 10](#_Toc213423611)

[References 13](#_Toc213423612)

# **Online Resource methods**

## Parameter calculations

Prevalence of AD dementia was calculated from published prevalence of all-cause dementia for males and females by age group^1,2^, assuming AD accounts for 65% of all dementia cases^3,4^. Similarly, prevalence and incidence of MCI due to AD were calculated from published prevalence^5,6^ and incidence^7^ of MCI due to AD by age group, then multiplied by the corresponding published percentages with amyloid abnormality^5^. As most studies investigated dementia and MCI due to AD in the older population, prevalence and incidence were fitted to other age groups using exponential transformation on the linear regression of prevalence and incidence on age group (Figures S1 – S3). People under 30 years of age were assumed to be completely free from MCI due to AD and AD dementia i.e. zero prevalence and incidence.

Sensitivity analyses were performed based on medium DMT efficacy (25%) with majority primary care follow-up using combinations of uptake, number of years to reach such uptake and proportion receiving primary care after initial specialist care. Values of the three parameters were based on expert responses (Table S1), using the extreme high, extreme low, weighted mean high and weighted mean low combinations (Table S2).

## Cost Equations

For each projection year, the annual healthcare costs, social care costs and additional treatment costs associated with DMT were calculated for each sex and age band and aggregated to give a total annual cost. Annual costs were summed across the 20-year projection period to calculate cumulative costs.

### Equation 1: Total cost equation for annual healthcare costs and annual social care costs for a given year, for each sex and age band:

$$C_{\tau}^{(T)}= C_{\tau}^{(G)}\times\left( P_{U}+P_{MCI} \right)+\sum_{i \in\{ADD states\}} C_{\tau}^{(AD)} \times\mu_{\tau}^{(i)} {\times P}_{i}$$

where:

$C_{\tau}=Cost of type \tau(where the type \tau is either healthcare or social care)$,

$$C_{\tau}^{\left( T \right)}=Total annual cost of type \tau,$$

$$C_{\tau}^{\left( G \right)}=Total annual cost of type \tau per person per year in England for the general population,$$

$$C_{\tau}^{\left( AD \right)}=Total annual cost of type \tau per person per year in England for the AD population,$$

$P_{U}= Total population in cognitively unimpaired state$,

$P_{MCI}= Total population in MCI due to AD state$,

$$P_{i}= Total population in ADD state i$$

$$\mu_{\tau}^{\left( i \right)}=AD severity cost ratio multiplier for cost type \tau and ADD state i,$$

$$\left\{ ADD states \right\}=\left\{ Mild AD dementia, Moderate AD dementia, Severe AD dementia \right\}.$$

### Equation 2: Total additional treatment costs associated with DMT (excluding the costs of the DMT itself) for each care model, for a given year for each sex and age band:

$$C_{t}^{\left( T \right)}=\alpha\times C_{t}^{\left( M \right)}{\times P}_{E}+C_{t}^{\left( S \right)} {\times N}_{E}$$

$$C_{t}^{(M)}=\sum_{j\in\{Care settings\}} \rho_{j}^{(M)} \times v_{j}^{(M)} {\times C}_{t}^{(j)}$$

where:

$$C_{t}^{\left( T \right)}=Total annual treatment cost associated with DMT,$$

$$C_{t}^{\left( M \right)}=Annual treatment cost associated with DMT for the given care model \left( majority primary or majority specialist \right),$$

$C_{t}^{\left( S \right)}=Cost of a single specialist care visit$,

$P_{E}=Treatment eligible population \left( MCI due to AD and Mild AD dementia populations \right)$,

$N_{E}=Newly treated population in the given year$,

$\alpha=Proportion of treatment eligible population with access to DMT$,

$\rho_{j}^{\left( M \right)}= Proportion of treated population receiving treatment in care setting j for the given care model$,

$v_{j}^{(M)}= Number of visits to care setting j for the given care model$,

$C_{t}^{\left( j \right)}=Cost of a single visit to care setting j$,

$\left\{ Care settings \right\}=\{Primary care, Specialist care\}$.

## Model inputs and analysis – further information

The baseline population projections underpinned the demographic changes from 2020-2040. The population estimate, along with baseline prevalence estimates were used to estimate state occupancy in 2020. In a given year, the age-specific transitions to MCI due to AD, and the subsequent AD dementia state transitions were modelled. The cognitively unimpaired population was then calculated as the difference between the population projections and the total number alive in the MCI due to AD and AD dementia states.

Four clinical and scientific experts, who are co-authors of this study, provided their insights on the key parameters of the model. Their names are listed below in alphabetical order:

- Craig Ritchie - Honorary Professor of Brain Health and Neurodegenerative Disease at the University of St Andrews, UK
- Dominic Trepel - Associate Professor of Psychiatry at the Trinity Institute of Neurosciences, Trinity College Dublin, Ireland
- Marc Evans - Consultant diabetologist at the University Hospital of Llandough, Cardiff, UK
- Sophie Edwards - Consultant in elderly medicine and clinical lead for older people’s services at The Royal London Hospital, London, UK

# **Online Resource figures**

## Figure S1. Dementia prevalence fitting for A) males and B) females^*^

A)


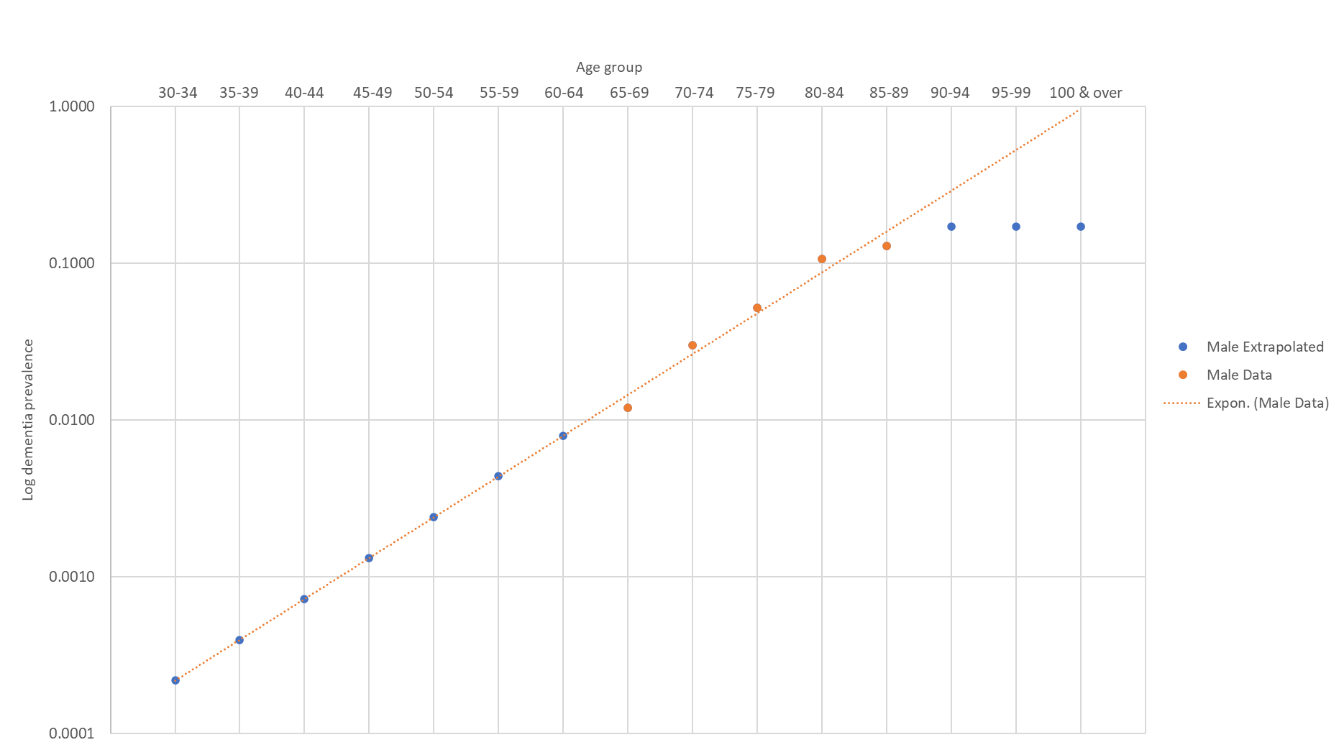


B)


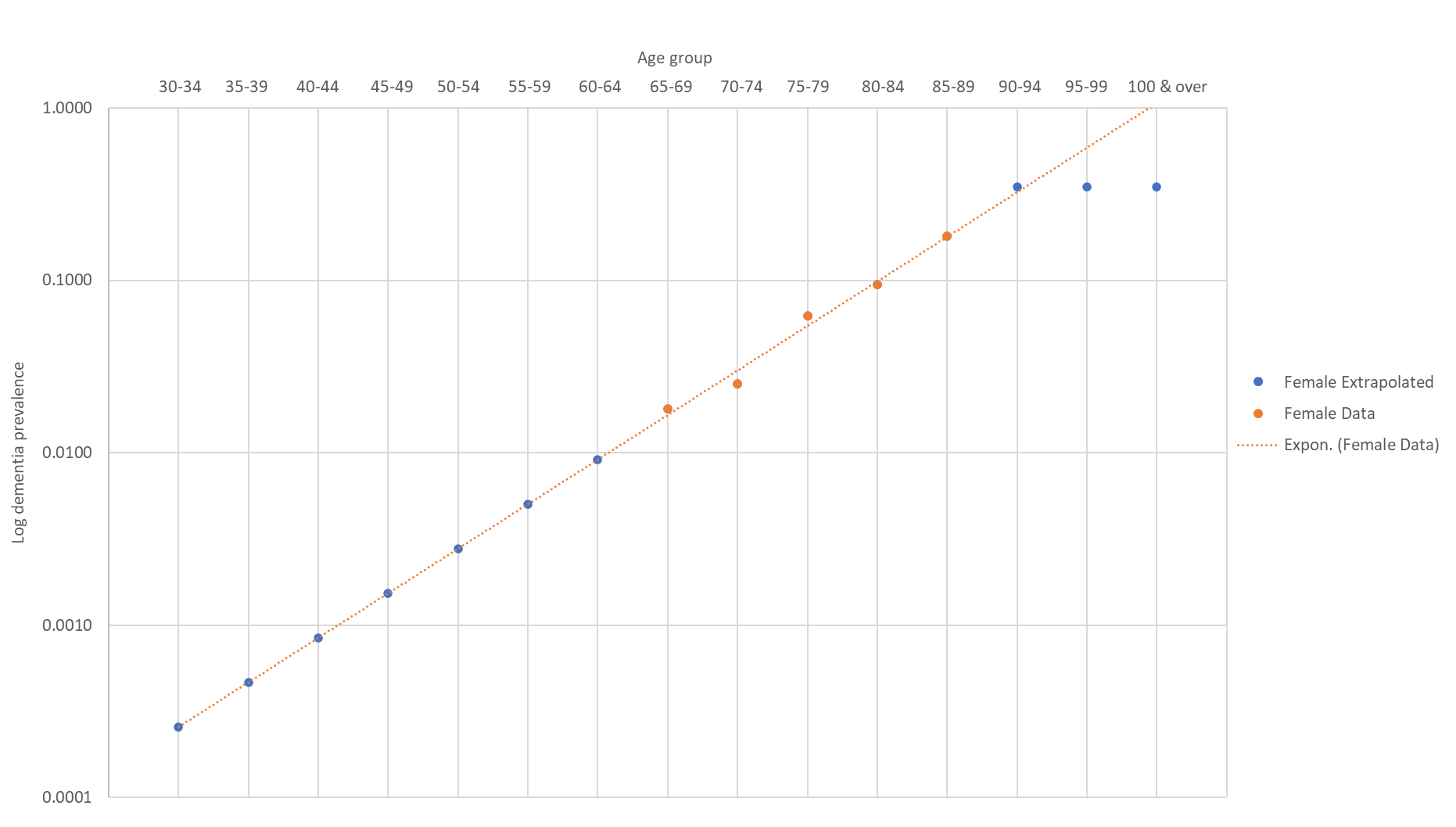


^*^ Dementia prevalence for age groups between 65 and 89 years of age from Matthews et al.^1^ and Wittenberg et al.^2^

## Figure S2. MCI due to AD prevalence fitting^†^


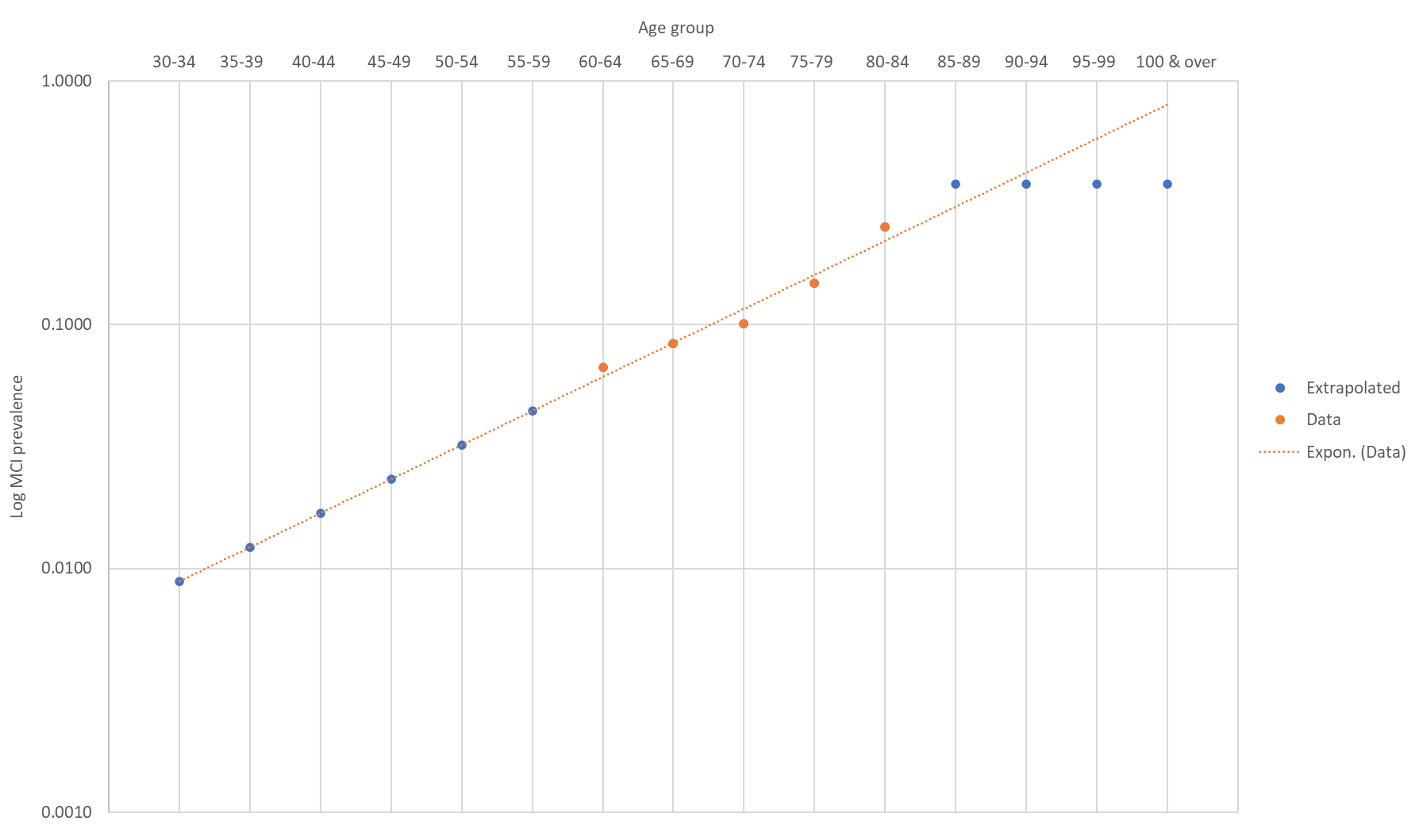


^†^ MCI prevalence for age groups between 60 and 84 years of age from Gabelle et al.^5^ and Petersen et al.^6^

## Figure S3. MCI due to AD incidence fitting^‡^


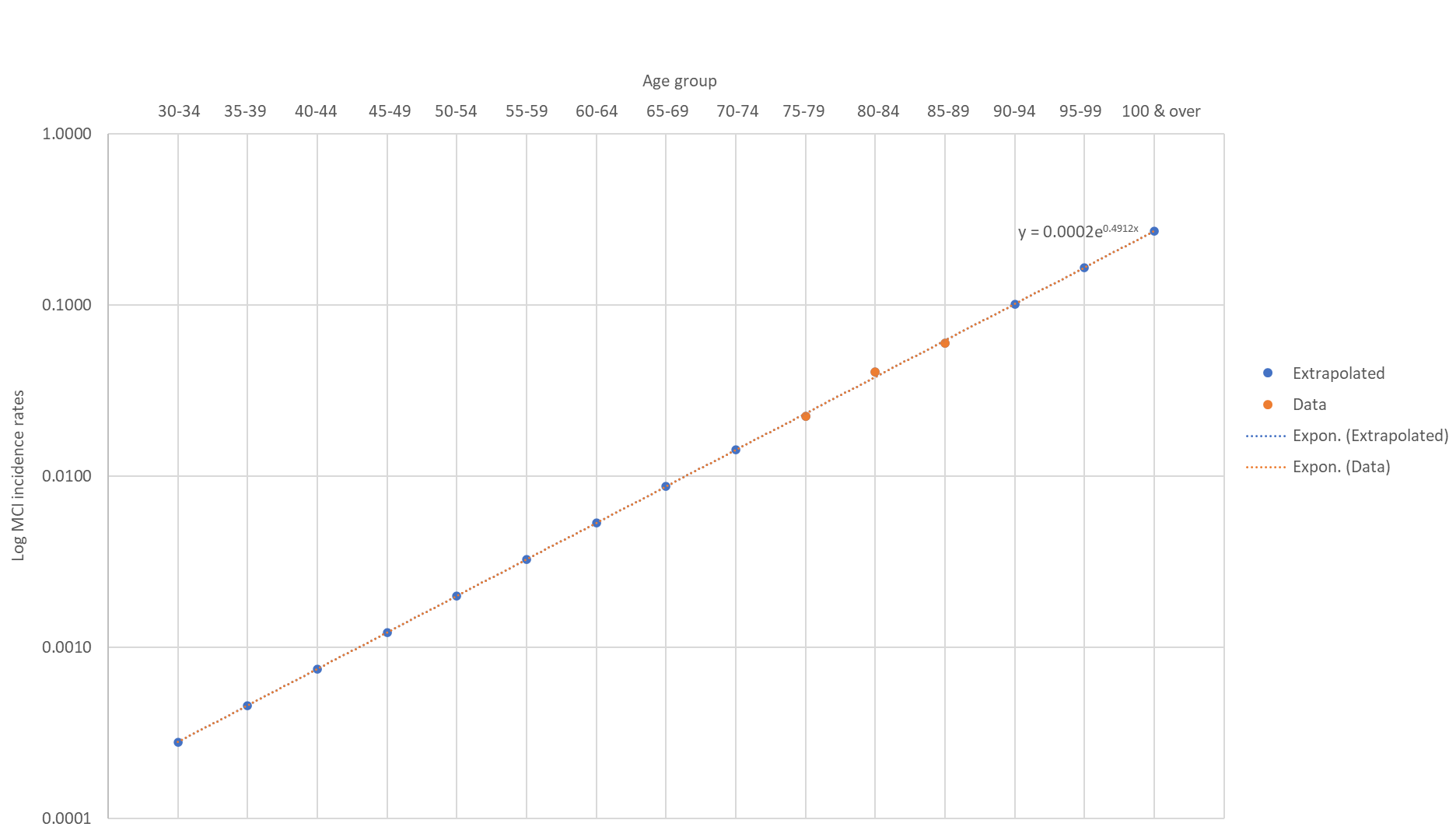


^‡^ MCI incidence for age groups between 75 and 89 years of age from Gillis et al.^7^

## Figure S4. Cumulative cost savings by 2040 versus no DMT in sensitivity analyses

# **Online Resource tables**

## Table S1. Consolidated expert responses

| **Question** | **Most likely value** | **Means of lowest and highest plausible values** | **Extremes of lowest and highest plausible values** |
| --- | --- | --- | --- |
| **High proportion of DMT treated** | 58% | 25-70% | 10-100% |
| **Low proportion of DMT treated** | 25% | 11-54% | 2-100% |
| **Number of years to reach high proportion** | 8.7 | 5.7-12.3 | 1-20 |
| **Majority primary care follow-up** | 86% | 50-88% | 20-100% |
| **Majority specialist care follow-up** | 56% | 20-70% | 0-100% |

**Abbreviations**: DMT, disease-modifying therapies;

## Table S2. Parameters for further scenario analysis

|  | **Extreme low values** | **Weighted mean low values** | **Weighted mean high values** | **Extreme high values** |
| --- | --- | --- | --- | --- |
| Uptake level | 10% | 25% | 70% | 100% |
| No. years to reach uptake level | 20 | 12 | 6 | 1 |
| Majority primary: % receiving primary care | 20% | 50% | 88% | 100% |
| Majority specialist: % receiving specialist care | 0% | 20% | 70% | 100% |

## Table S3. Total AD population eligible for DMT and receiving DMT from 2020-2040; irrespective of care model

| **Year** | **2020** | **2025** | **2030** | **2035** | **2040** |
| --- | --- | --- | --- | --- | --- |
| **Total AD population eligible for treatment (MCI due to AD or Mild AD dementia)** | | | | | |
| No DMT | 1,766,428 | 2,296,315 | 2,679,363 | 3,027,321 | 3,355,730 |
| High uptake, High efficacy | 1,766,428 | 2,296,315 | 2,701,829 | 3,112,693 | 3,510,868 |
| High uptake, Medium efficacy | 1,766,428 | 2,296,315 | 2,698,097 | 3,098,511 | 3,484,840 |
| High uptake, Low efficacy | 1,766,428 | 2,296,315 | 2,694,360 | 3,084,307 | 3,458,868 |
| Low uptake, Medium efficacy | 1,766,428 | 2,296,315 | 2,687,524 | 3,058,320 | 3,411,617 |
| **Total population receiving DMT** | | | | | |
| High uptake, High efficacy | 0 | 146,709 | 1,035,583 | 1,789,798 | 2,018,749 |
| High uptake, Medium efficacy | 0 | 146,709 | 1,034,153 | 1,781,644 | 2,003,783 |
| High uptake, Low efficacy | 0 | 146,709 | 1,032,720 | 1,773,476 | 1,988,849 |
| Low uptake, Medium efficacy | 0 | 63,787 | 447,870 | 764,580 | 852,904 |

## Table S4. Annual costs of DMT in 2040 across uptake and efficacy scenarios.

| **Scenario** | **Annual DMT cost in 2040** | | |
| --- | --- | --- | --- |
|  | **Low cost** | **Medium cost** | **High cost** |
| No DMT | £0.0bn | £0.0bn | £0.0bn |
| High uptake, High efficacy | £2.4bn | £22.4bn | £42.4bn |
| High uptake, Medium efficacy | £2.4bn | £22.2bn | £42.1bn |
| High uptake, Low efficacy | £2.4bn | £22.1bn | £41.8bn |
| Low uptake, Medium efficacy | £1.0bn | £9.5bn | £17.9bn |

# **CHEERS 2022 Checklist**

| **Topic** | **No.** | **Item** | **Location where item is reported** |
| --- | --- | --- | --- |
| **Title** |  |  |  |
|  | 1 | Identify the study as an economic evaluation and specify the interventions being compared. | Title, Page 2.  Scenario Modelling study |
| **Abstract** |  |  |  |
|  | 2 | Provide a structured summary that highlights context, key methods, results, and alternative analyses. | Abstract, Page 2 and Key messages page 3 |
| **Introduction** |  |  |  |
| **Background and objectives** | 3 | Give the context for the study, the study question, and its practical relevance for decision making in policy or practice. | Introduction, Page 4, paragraph 4 |
| **Methods** |  |  |  |
| **Health economic analysis plan** | 4 | Indicate whether a health economic analysis plan was developed and where available. | Not Reported |
| **Study population** | 5 | Describe characteristics of the study population (such as age range, demographics, socioeconomic, or clinical characteristics). | Methods, Page 4, Model structure and parameters for baseline scenario  Key baseline parameters Table 1 and Supplementary Table S1 |
| **Setting and location** | 6 | Provide relevant contextual information that may influence findings. | Introduction, Page 4 |
| **Comparators** | 7 | Describe the interventions or strategies being compared and why chosen. | Methods, Page 5, Model Inputs: Key treatment efficacy, uptake, and care model parameters influencing AD populations |
| **Perspective** | 8 | State the perspective(s) adopted by the study and why chosen. | Methods, Page 5, Model Inputs: Healthcare, social care, and treatment costs |
| **Time horizon** | 9 | State the time horizon for the study and why appropriate. | Methods, Page 4, Model structure |
| **Discount rate** | 10 | Report the discount rate(s) and reason chosen. | Methods, Page 5, Model Inputs: Healthcare, social care, and treatment costs |
| **Selection of outcomes** | 11 | Describe what outcomes were used as the measure(s) of benefit(s) and harm(s). | Methods, Page 6, Model analysis |
| **Measurement of outcomes** | 12 | Describe how outcomes used to capture benefit(s) and harm(s) were measured. | Not Applicable |
| **Valuation of outcomes** | 13 | Describe the population and methods used to measure and value outcomes. | Not Applicable |
| **Measurement and valuation of resources and costs** | 14 | Describe how costs were valued. | Methods, Page 5, Model Inputs: Healthcare, social care, and treatment costs |
| **Currency, price date, and conversion** | 15 | Report the dates of the estimated resource quantities and unit costs, plus the currency and year of conversion. | Methods, Page 6, Model analysis. Table 1. |
| **Rationale and description of model** | 16 | If modelling is used, describe in detail and why used. Report if the model is publicly available and where it can be accessed. | Methods, Page 4, Model Structure,  Methods, Page 5, Model inputs: Parameters for baseline scenario  Supplementary material |
| **Analytics and assumptions** | 17 | Describe any methods for analysing or statistically transforming data, any extrapolation methods, and approaches for validating any model used. | Methods, Page 5, Model Inputs,  Discussion, Page 7,  Supplementary material |
| **Characterising heterogeneity** | 18 | Describe any methods used for estimating how the results of the study vary for subgroups. | Not Applicable |
| **Characterising distributional effects** | 19 | Describe how impacts are distributed across different individuals or adjustments made to reflect priority populations. | Not Applicable |
| **Characterising uncertainty** | 20 | Describe methods to characterise any sources of uncertainty in the analysis. | Methods, Page 6, Model analysis |
| **Approach to engagement with patients and others affected by the study** | 21 | Describe any approaches to engage patients or service recipients, the general public, communities, or stakeholders (such as clinicians or payers) in the design of the study. | Methods, Page 5, Model inputs: Key treatment efficacy, uptake, and care model parameters influencing AD populations  Supplementary material, table S6 |
| **Results** |  |  |  |
| **Study parameters** | 22 | Report all analytic inputs (such as values, ranges, references) including uncertainty or distributional assumptions. | Table 1, Page 12  Figure 1, Page 14  Supplementary tables. |
| **Summary of main results** | 23 | Report the mean values for the main categories of costs and outcomes of interest and summarise them in the most appropriate overall measure. | Results, Page 6, paragraph 1-3  Table 2, Page 13  Figure 4, Page 17 |
| **Effect of uncertainty** | 24 | Describe how uncertainty about analytic judgments, inputs, or projections affect findings. Report the effect of choice of discount rate and time horizon, if applicable. | Results, Page 6, paragraph 4 |
| **Effect of engagement with patients and others affected by the study** | 25 | Report on any difference patient/service recipient, general public, community, or stakeholder involvement made to the approach or findings of the study | Not reported |
| **Discussion** |  |  |  |
| **Study findings, limitations, generalisability, and current knowledge** | 26 | Report key findings, limitations, ethical or equity considerations not captured, and how these could affect patients, policy, or practice. | Discussion, Page 7-8 |
| **Other relevant information** |  |  |  |
| **Source of funding** | 27 | Describe how the study was funded and any role of the funder in the identification, design, conduct, and reporting of the analysis | End of manuscript, Page 9 |
| **Conflicts of interest** | 28 | Report authors conflicts of interest according to journal or International Committee of Medical Journal Editors requirements. | End of manuscript, Page 9 |

*From:* Husereau D, Drummond M, Augustovski F, et al. Consolidated Health Economic Evaluation Reporting Standards 2022 (CHEERS 2022) Explanation and Elaboration: A Report of the ISPOR CHEERS II Good Practices Task Force. Value Health 2022;25. <doi:10.1016/j.jval.2021.10.008>

# References

1. Matthews FE, Arthur A, Barnes LE, Bond J, Jagger C, Robinson L, et al. A two-decade comparison of prevalence of dementia in individuals aged 65 years and older from three geographical areas of England: results of the Cognitive Function and Ageing Study I and II. The Lancet. 2013 Oct 26;382(9902):1405–12.

2. Wittenberg R, Hu B, Barraza-Araiza L, Rehill A. Projections of older people with dementia and costs of dementia care in the United Kingdom, 2019–2040. London: London School of Economics;

3. Alzheimer’s Society. Alzheimer’s Society’s view on demography [Internet]. [cited 2023 Apr 5]. Available from: https://www.alzheimers.org.uk/about-us/policy-and-influencing/what-we-think/demography

4. World Health Organization. Dementia fact sheet [Internet]. [cited 2023 Apr 5]. Available from: https://www.who.int/news-room/fact-sheets/detail/dementia

5. Gabelle A, Guéry M, Doutriaux A, Bettayeb K. Forecasting the Prevalence of Alzheimer’s Disease at Mild Cognitive Impairment and Mild Dementia Stages in France in 2022. J Prev Alzheimers Dis. 2023 Apr 1;10(2):259–66.

6. Petersen RC, Lopez O, Armstrong MJ, Getchius TSD, Ganguli M, Gloss D, et al. Practice guideline update summary: Mild cognitive impairment. Neurology. 2018 Jan 16;90(3):126–35.

7. Gillis C, Mirzaei F, Potashman M, Ikram MA, Maserejian N. The incidence of mild cognitive impairment: A systematic review and data synthesis. Alzheimers Dement Diagn Assess Dis Monit. 2019 Mar 8;11:248–56.
